# Foot thermometry with mHeath-based supplementation to prevent diabetic foot ulcers: A randomized controlled trial

**DOI:** 10.12688/wellcomeopenres.15531.2

**Published:** 2020-08-28

**Authors:** Maria Lazo-Porras, Antonio Bernabe-Ortiz, Alvaro Taype-Rondan, Robert H. Gilman, German Malaga, Helard Manrique, Luis Neyra, Jorge Calderon, Miguel Pinto, David G. Armstrong, Victor M. Montori, J. Jaime Miranda

**Affiliations:** 1 CRONICAS Centre of Excellence in Chronic Diseases, Universidad Peruana Cayetano Heredia, Lima, Peru; 2 School of Public Health and Administration, Universidad Peruana Cayetano Heredia, Lima, Peru; 3 Department of International Health, Johns Hopkins Bloomberg School of Public Health, Baltimore, Maryland, USA; 4 Área de Investigación y Desarrollo, Asociación Benéfica PRISMA, Lima, Peru; 5 Department of Medicine, School of Medicine, Universidad Peruana Cayetano Heredia, Lima, Peru; 6 Endocrinology Service, Hospital Nacional Arzobispo Loayza, Lima, Peru; 7 Endocrinology Service, Hospital Cayetano Heredia, Lima, Peru; 8 Southwestern Academic Limb Salvage Alliance (SALSA), Department of Surgery, Keck School of Medicine, University of Southern California, Los Angeles, California, USA; 9 Knowledge and Evaluation Research Unit, Mayo Clinic, Rochester, Minnesota, USA

**Keywords:** type 2 diabetes mellitus, diabetic foot ulcer, prevention, implementation, mHealth

## Abstract

**Background**: Novel approaches to reduce diabetic foot ulcers (DFU) in low- and middle-income countries are needed. Our objective was to compare incidence of DFUs in the thermometry plus mobile health (mHealth) reminders (intervention) vs. thermometry-only (control).

**Methods**: We conducted a randomized trial enrolling adults with type 2 diabetes mellitus at risk of foot ulcers (risk groups 2 or 3) but without foot ulcers at the time of recruitment, and allocating them to control (instruction to use a liquid crystal-based foot thermometer daily) or intervention (same instruction supplemented with text and voice messages with reminders to use the device and messages to promote foot care) groups, and followed for 18 months. The primary outcome was time to occurrence of DFU. A process evaluation was also conducted.

**Results**: A total of 172 patients (63% women, mean age 61 years) were enrolled; 86 to each study group. More patients enrolled in the intervention arm had a history of previous DFU (66% vs. 48%). Follow-up for the primary endpoint was complete for 158 of 172 participants (92%). Adherence to ≥80% of daily temperature measurements was 87% (103 of 118) among the study participants who returned the logbook. DFU cumulative incidence was 24% (19 of 79) in the intervention arm and 11% (9 of 79) in the control arm. After adjusting for history of foot ulceration and study site, the hazard ratio (HR) for DFU was 1.44 (95% CI 0.65, 3.22).

**Conclusions**: In our study, conducted in a low-income setting, the addition of mHealth to foot thermometry was not effective in reducing foot ulceration. Importantly, there was a higher rate of previous DFU in the intervention group, the adherence to thermometry was high, and the expected rates of DFU used in our sample size calculations were not met.

**Trial registration**: ClinicalTrials.gov NCT02373592 (27/02/2015)

## Background

The prevalence of type 2 diabetes mellitus in the adult population worldwide has doubled from 4.7% in 1980 to 8.5% in 2014 ^1^. Low- and middle-income countries (LMICs) are disproportionally affected by diabetes, since diabetes-related complications, such as diabetic foot ulcer (DFU), are more frequent in these contexts ^1, 2^. In the US, 60%–70% of people with diabetes will develop peripheral neuropathy ^3^. This is important since one in four patients with peripheral neuropathy will develop a DFU, which will increase the risk of foot amputation significantly ^4^.

Thermometry is a tool that can identify early signs of foot inflammation, thus providing early signals to enact management and reduce the incidence of DFU and amputation ^5^. Three previous clinical trials ^6– 8^ and one systematic review have found that the use of thermometry reduced DFU incidence four- to ten-fold among individuals with diabetes at high-risk of developing a DFU ^9^. Additionally, one study found that the addition of counselling to promote self-monitoring of skin temperature to standard care is feasible ^10^. However, the benefits of thermometry depend on patient adherence to self-assessment, and foot temperature should be evaluated on at least half of the days to effectively reduce the risk of foot ulceration ^7^. Yet, adherence could be challenging, especially in LMIC settings. Therefore, novel approaches to improve self-management thermometry adherence are needed. In this context, interventions using short message service (SMS) for diabetes management have been found to be useful to improve self-efficacy, social support ^11^, and clinical diabetes-related outcomes ^12^.

Other approaches that could prevent foot ulcers include patient’s foot self-care behaviour, annual foot evaluations, knowledge about diabetic foot in health care workers, and therapeutic footwear ^13^. Also, in order to prevent recurrent ulcers, it is important to consider the integration or combination of these approaches ^14^.

We propose to evaluate the efficacy of a combination of foot thermometry plus mobile health (mHealth)-delivered reminders, using SMS and voice messaging, in reducing DFU in Peru. Our objective was to compare incidence of DFU in the thermometry plus mHealth reminders intervention arm vs. thermometry-only control arm.

## Methods

### Trial design

This was a physician- and evaluator-blinded, 18-month, randomized clinical trial with two parallel arms and a 1:1 allocation. Details of the intervention and the study protocol have been published elsewhere ^15^. We followed the extension of the CONSORT 2010 statement for reporting pragmatic trials ^16^.

Although initially planned to follow participants for 12 months, we decided to extent the follow-up period to 18 months to accrue enough DFU events, as we noticed that the frequency of DFU at six months was lower than we expected. Thus, only the extension of the trial follow-up was changed without affecting randomization or assessment rates. There were no other deviations from the original trial protocol.

### Participants

Participants were recruited at the outpatient clinics of two third-level public hospitals in Lima, Peru; Hospital Nacional Cayetano Heredia and Hospital Nacional Arzobispo Loayza. In some cases, physicians referred the patient to the study fieldworkers to perform a foot evaluation and in other cases fieldworkers conducted an active search for potential participants in the waiting room of the Endocrinology clinic.

Patients were eligible if they: had a diagnosis of type 2 diabetes mellitus; were between 18 and 80 years of age; were in risk group 2 or 3 using the diabetic foot risk classification system as specified by the International Working Group on the Diabetic Foot ([IWGDF], neuropathy and deformity = category 2, history of ulcer and/or amputation = category 3) ^17– 19^; had a palpable dorsalis pedis pulse in both feet; had an operating cell phone or a caregiver with an operating cell phone; and had the ability to provide informed consent. Patients were considered not eligible if they had current foot ulcers, active Charcot osteoarthropathy, severe peripheral arterial disease, or foot infection.

Our eligibility criteria used IWGDF categories and included people with diabetes at risk of ulceration group 2 and 3. In so doing, rather than focusing only on those at the highest risk for ulceration (IWGDF group 3) we wanted to pursue a pragmatic approach for the prevention of DFU among people with diabetes, thus including also those participants from the IWGDF group 2 category. All previous studies included mostly participants from IWGDF group 3, and only one clinical trial included group 2 patients.

### Development and validation of mHealth messages

The content of the mHealth messages was developed and validated with 19 people with type 2 diabetes mellitus. Messages were tested using short open surveys to evaluate the clarity and appropriateness of the messages. These messages were constructed based on a literature review about the characteristics of health education messages, paired with the advice from a specialist in health communication, taking into consideration the reading level of our population and the use of short messages focused on a single idea. We also asked colleagues with previous experience on the use of SMS and mHealth to review the messages before testing them with patients, and changes were introduced after their revision.

We printed all the messages in a single page which was provided to the participants to read by himself/herself. Afterwards, we evaluated each message using the following six questions: (1) Is the message clear?, (2) Could you tell me how would you explain the content of the message to another person?, (3) Is there any word(s) that is difficult to understand?, (4) Is there something that you do not like about the message?, (5) Is there any suggestion to improve the message?, and (6) Would you prefer to be addressed in a formal way “usted” or an informal way “tu”? (see *Extended data* ^20^).

### Interventions

**Figure 1.**
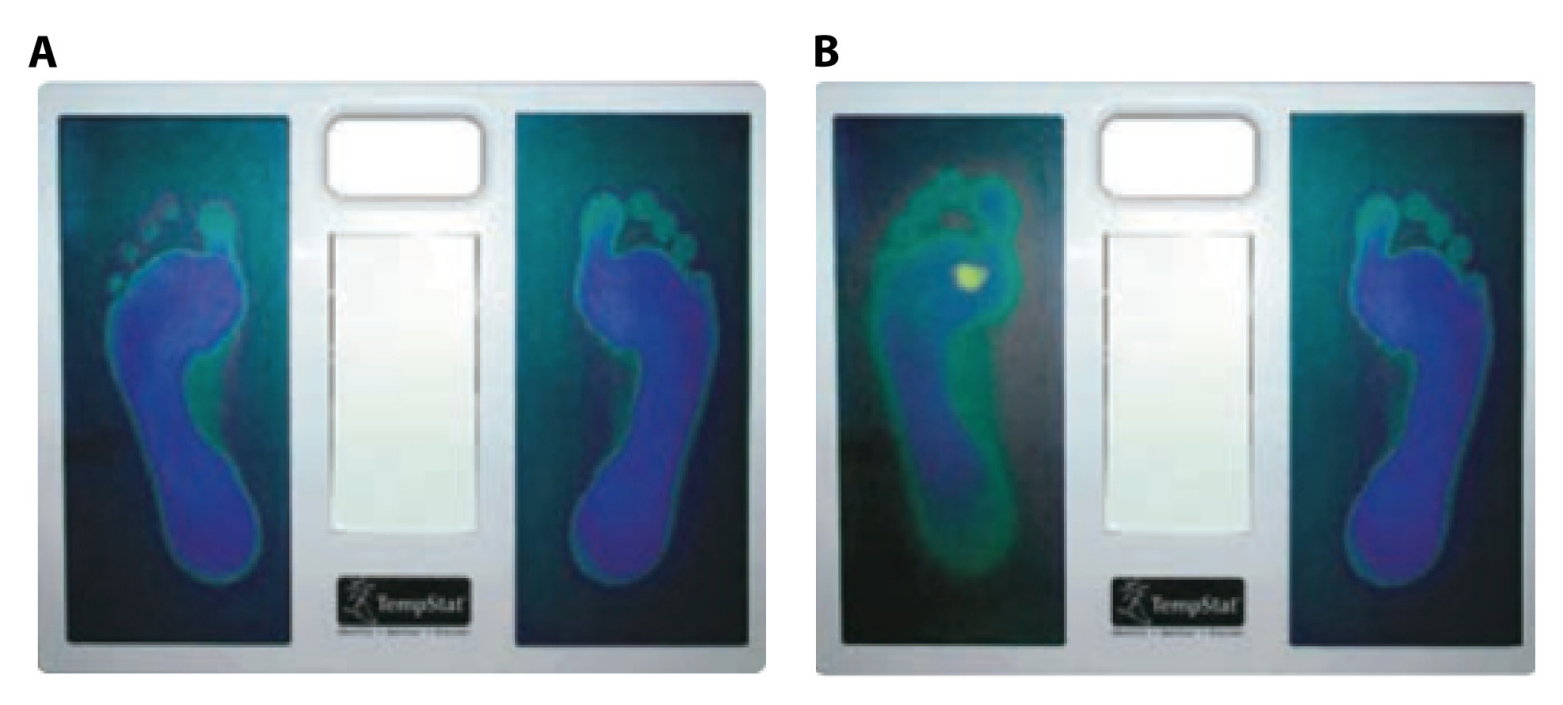
TempStat. **A**) Normal appearance. **B**) Alarm sign (yellow spot). Source: Visual Footcare Technologies LLC ©, 2013.

At the initiation visit, all participants received education about foot care, i.e. etiology and risk factors for the development of neuropathy and ulcers, as well as recommendations for foot care practices and early signs of ulceration; and instructions for the use of the TempStat™ device (see *Extended data*). This foot care education was done through three videos that were validated by physicians and patients with type 2 diabetes mellitus. The first two videos lasted 8 and 6 minutes and they were related to foot care, whereas the third video lasted 6 minutes and presented the instructions on the use of the TempStat™ device. The three videos were in Spanish and were showed once at the initiation visit, as detailed elsewhere ^15^. The device uses liquid crystal technology to provide a visual image of the temperatures (e.g. yellow image represents a higher temperature than blue image) ( Figure 1). Frykberg *et al*. ^21^ showed that TempStat™ can detect alarm signs, represented by a yellow color change, and the results positively correlate to temperature findings of infrared thermometer, the gold standard of thermometry devices. Another study found that the device identified 74% of serious foot problems ^22^.

One week after enrollment, the TempStat™ was provided to each participant. Fieldworkers instructed the participants to use the device daily and to contact them by phone or SMS if one of the alarm signs appeared in the pads of the TempStat™: two different colors in the contralateral areas of the feet or a yellow spot in any area for two consecutive days. In these cases, the nurse asked about any lesions in the feet as well as the participant’s activity in the last two weeks and provided recommendations on how to decrease activity until foot temperature normalized. Also, in cases where the alarm sign persisted more than one week, an in-person evaluation was performed to assess the patient for infection and/or a masked injury. Additionally, participants were trained to contact the study nurse in cases of dermal lesion of the foot and they were asked to be evaluated promptly by a nurse who was blind to the intervention. When a DFU was confirmed, the study nurse referred the patients to follow the standard protocol.

In the intervention arm, in addition to the TempStat™, participants received the mHealth component weekly (two reminder messages and six foot-care promotion messages each week) for the 18-month study period via both SMS and voice messaging.

Developed and validated messages ^23^ were sent at 8am approximately and, for the first two weeks of the intervention, daily (Monday to Friday) reminders to use the TempStat™ were sent. Thereafter, for the remaining 76 weeks, patients received only two messages per week at the same time: the content alternating between reminders to use the TempStat™ and promotion of foot care (one SMS and one voice message). Messages were delivered to the participant or caregiver’s cell phones through an automated software system developed by the study team (see *Software availability* ^24^). Every week the system was evaluated by the study coordinator to verify its functionality.

### Study procedures

At baseline, enrolled participants provided information to the fieldworker through questionnaires on lifestyle, history of cardiovascular disease and diabetes, current diabetes treatment, use of insoles, use of orthopedic shoes and mobile phone literacy and underwent a demographic evaluation (age, gender, educational level), socioeconomic evaluation (working status), depression assessment (Patient Health Questionnaire-9), anthropometric evaluation (weight, height and body mass index) and blood pressure measurements (see *Extended data* ^25^).

Periodic assessments of the participants involving a general checkup and lower extremity evaluation was conducted every two months by the nurse evaluator. Additionally, the nurse collected data about diabetes treatment, caregiver presence, use of insoles and/or orthopedic shoes, and had their weight and blood pressure measured ( *Extended data* ^25^). In some cases, participants could not attend to the hospital for the checkup; in those cases, we completed the visit by phone or by domiciliary visits. In the last visit at 18 months, participants were asked to return their logbook of temperature measurements. In general, participants were encouraged to maintain regular visits with their treating physician in the outpatient clinic.

Glycated hemoglobin (HbA1c) was measured at baseline, six, 12 and 18 months. Measurements at baseline and 18 months were used for the study and measurements at six and 12 months were for standard of care. HbA1c was measured using high-performance liquid chromatography (D10, BioRad, Munich, Germany). The blood sample was collected in the endocrinology clinic by the nurse evaluator during the periodic assessment at the time periods specify above. All samples were transported to be analyzed in a single facility and were checked with regular external standards and internal duplicate assays and monitored by BioRad for quality control.

### Outcomes

The primary outcome was DFU. The definition was based on the American Diabetes Association criteria ^26, 27^ and for this study it was considered as the presence of DFU occurring at any point during the 18-month study period after randomization. The evaluator was a trained nurse blind to the intervention allocation. The identification of a DFU was through three ways: during the bimonthly clinical nurse evaluations; if an alarm sign had been noted and prompted the participant to seek clinical evaluation; or if the participant identifies a dermal lesion and seeks clinical evaluation.

The following were pre-defined as secondary outcomes: adherence to daily temperature measurement, defined as the participants having recorded their temperature measurements in the logbook on ≥80% of days, and ≥1% reduction in HbA1c when comparing the 18-month with baseline values. Another outcome was alarm signs registered in the logbook.

Our protocol ^15^ considered one additional pre-defined secondary outcomes: frequency of alarm signs reported to the study nurse. This was not analyzed because of their low frequency. The dose-response analysis of SMS and voice messaging, pre-specified as a secondary outcome in the protocol, was included as part of the process evaluation.

### Sub-group analyses

Our *a priori* sub-group analyses were i) previous foot ulceration and ii) caregiving status, considering assistance provided to the patient with basic activities of daily living, or in the identification, prevention, or treatment of diabetes or any disability. Also, within the intervention-arm only, the type of recipient of the messaging (patient vs. caregivers) was considered for sub-group analyses. In our protocol ^15^, we also considered sub-group analyses of participants that use insoles and/or orthopedic shoes, but these were not analyzed due to low frequency.

### Sample size

The sample size was estimated using data from previous randomized trials in study populations similar to our study population ^7, 8^. We expected an absolute change of 21% between the intervention arm and the control arm (9% vs 30%) and with a power of 0.9 and an alpha of 0.05, we required a sample size of 78 participants. We planned to enroll 86 participants in each study arm, anticipating a 10% dropout rate.

### Randomization

We conducted stratification using the hospital site as a single stratum and blocks of 6 to generate a random allocation sequence. Sealed envelopes with codes to randomize participants were used. An independent researcher prepared the envelopes, and the study nurses assigned the codes to each of the enrolled participants. Separately, the study coordinator was responsible for opening the envelopes and informing participants about their intervention or control allocation as per the random list. The nurse/independent evaluators were not aware of the patient's group allocation.

### Blinding

The participants were instructed not to discuss their treatment assignment with the blinded evaluator. Physicians providing care to study participants, nurses and the field coordinators were blind to treatment allocation.

### Process evaluation

Additionally, we performed a process evaluation during the 18-month follow-up visit to a random group of participants of the two study sites. We obtained information through a set of questions and direct observation of the use of the TempStat™ with 102 participants. In addition, with 39 participants, we asked close and open questions about the messages received in the week prior to the 18-month follow-up visit. As part of this process evaluation, we aimed to know: i) if participants knew how to use the TempStat™; ii) how many SMS and voice messages were delivered by the automated system to study participants according to the automated system; iii) how many SMS and voice messages were received by study participants according to the automated system; iv) if participants understood the messages (only if participants reported that they had received a message in the previous two weeks); and v) opinions from the participants about their preferences in SMS vs. voice messages.

The process evaluation was performed by two fieldworkers different to those who delivered the intervention and data collection was conducted through observation (participants were asked to show how they used the TempStat™), questionnaire (about nursing consultation, report of communication with study nurses, reasons for communication, alarm sign detection) and open questions (related to SMS or voice messaging preferences, use of TempStat™, suggestions about how to improve the intervention) ^25^.

### Statistical methods

To compare the rates of DFU between study arms we performed a time-to-event approximation using Cox’s regression, having time to DFU at 18 months as an outcome. Hazard ratios (HR) and their respective 95% confidence intervals (95% CI) were estimated for the primary outcome of DFU and for the *a priori* defined sub-group analyses. These analyses included all retained participants, regardless of the number of visits attended, following the intention-to-treat principle. The model was adjusted by site and history of previous ulcer. Evaluation of secondary outcomes of interest was performed using logistic regression analysis to calculate odds ratios (OR) and 95% CI. Data analysis was conducted in STATA V.14.0 (StataCorp, College Station, TX, USA).

For the process evaluation, frequencies and percentages are presented. Also, open-ended questions were transcribed, and then a codebook was created, themes were derived from the data. Coding was performed manually and patterns of answers are described.

### Ethics

The study protocol, informed consent templates, and questionnaires were reviewed and approved by the Institutional Review Board (IRB) at Universidad Peruana Cayetano Heredia (UPCH) in Lima, Peru (SIDISI 61482). In addition, participating hospitals (Hospital Cayetano Heredia and Hospital Nacional Arzobispo Loayza) in the study received the protocol and consent form for approval ^16^. The extension in the follow-up period was also approved by the IRB at UPCH and the participants re-consented. The fieldworker explained the study procedures, then the potential participant read the informed consent form and asked questions. After that, if they accepted, they signed the informed consent form. The trial was registered at ClinicalTrials.gov with the identifier NCT02373592 (27/02/2015).

## Results

The recruitment was conducted between October 2015 and March 2016 and the follow-up period lasted until October 2017.

**Figure 2.**
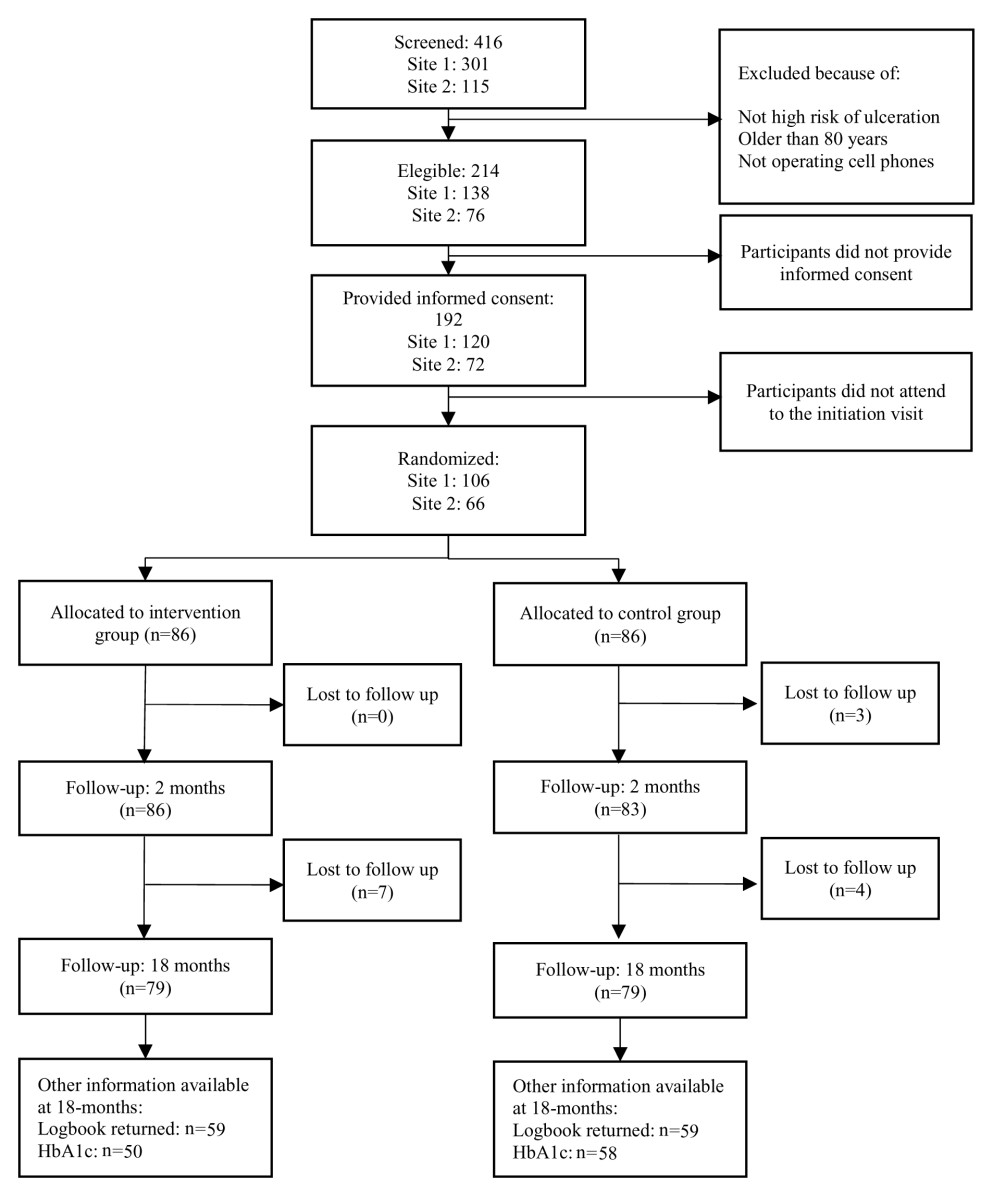
Flowchart.

In total, 416 participants were screened and 214 were eligible for the study. Of these, 192 gave informed consent and 172 attended the initiation visit and were allocated to the control (n=86) or intervention (n=86) arms ( Figure 2). Only 79/86 (91.9%) participants in each arm completed the 18-month follow-up. Reasons for lost to follow-up included migration back to the participant’s place of origin, wrong/incomplete addresses provided, or the participant did not answer the contact phone calls.

### Baseline characteristics

The baseline characteristics were similar between the intervention and control arms, with few exceptions ( Table 1). History of previous foot ulcers was reported with more frequency in the intervention arm; 65.9% vs. 48.2% in the control arm (p-value 0.02). Mean HbA1c was 8.9% in the intervention arm and 8.2% among the controls (p-value 0.03). In terms of mHealth literacy, there were no major differences between study arms, with the exception that participants in the intervention arm reported more frequently never having problems with cellphone coverage (89.5% vs. 74.4% in the control arm, p-value 0.01).

**Table 1.** Baseline characteristics.

	Control arm	Intervention arm
	(N=86) n (%)	(N=86) n (%)
**Site**		
Site 1	53 (61.6)	53 (61.6)
Site 2	33 (38.4)	33 (38.4)
**Sociodemographic variables**		
Age, mean (SD) *	62.1 (9.8)	60.3 (9.2)
Sex (female)	56 (65.1)	52 (60.5)
Level of education		
<7 years	30 (34.9)	30 (34.9)
7 to 11 years	40 (46.5)	42 (48.8)
12 or more years	16 (18.6)	14 (16.3)
Marital status: married or cohabitant	63 (73.3)	59 (68.6)
Currently working	34 (39.5)	33 (38.4)
Had a caregiver	35 (40.7)	36 (41.9)
**Clinical variables**		
Body mass index, mean (SD)	27.9 (4.8)	28.0 (4.4)
Depression (>9 points in PHQ-9)	23 (27.1)	22 (25.6)
Co-morbidities		
Hypertension diagnosis	37 (43.0)	41 (47.7)
Previous myocardial infarction	3 (3.5)	4 (4.7)
Other cardiac problems	3 (3.5)	3 (3.5)
Previous stroke	3 (3.5)	5 (5.8)
High cholesterol	48 (55.8)	41 (47.7)
**Behavioral variables**		
Current smoker (self- reported)	4 (4.7)	11 (12.9)
Binge drinking at least once during the last year	28 (32.6)	20 (23.3)
Physical activity (moderate/vigorous, three or more days a week)	7 (8.2)	13 (15.3)
**Variables related to** **diabetes**		
Years since diabetes diagnosis, mean (SD) *	12.7 (7.9)	13.3 (8.5)
HbA1c at baseline %, mean (SD)	8.2 (1.9)	8.9 (2.3)
Current pharmacological treatment for diabetes		
Metformin	67 (77.9)	72 (83.7)
Insulin	35 (40.7)	47 (54.7)
Consultations in the last 12 months		
Ophthalmology	48 (56.5)	45 (52.3)
Nephrology	16 (18.6)	21 (24.4)
Cardiology	30 (35.7)	35 (41.2)
Complications		
Diabetic retinopathy	13 (15.3)	21 (24.4)
Diabetic nephropathy	5 (6.0)	9 (10.6)
Hospitalization in the last year due to diabetes	10 (11.6)	9 (10.5)
Current use of orthopedic shoes **	0 (0)	4 (4.7)
Current use of insoles **	2 (2.3)	8 (9.3)
**mHealth literacy**		
The patient receives messages (instead than the caregiver)	45 (52.3)	45 (52.3)
The patient knows how to make calls **	81 (96.4)	85 (98.8)
The patient knows how to answer to calls **	82 (97.6)	85 (100.0)
The patient knows how to send SMS	77 (91.7)	75 (89.3)
The patient knows how to read SMS **	82 (97.6)	78 (91.8)
Never have problems with cellphone coverage	61 (74.4)	77 (89.5)
**Foot examination**		
Previous foot ulcers	40 (48.2)	56 (65.9)
Previous foot amputation	10 (12.1)	14 (16.5)
Any deformity in foot	53 (63.9)	54 (63.5)
Any alteration in monophilament test	71 (85.5)	70 (82.4)
Any alteration in biotensiometer (≥25)	65 (78.3)	75 (88.2)

* T-test; **Fisher’s exact text.

SD, standard deviation; PHQ, patient health questionnaire; mHealth, mobile health; SMS, short message service.

### Primary outcome

The cumulative incidence of DFU in the entire sample was 17.7% (28/158), and it was higher among participants with a history of previous ulceration (27.8%, 25/90) ^28^.

The incidence of DFU was 11.4% (95% CI 5.2% – 21.6%) in the control arm and 24.1% (95% CI 14.5% – 37.6%) in the intervention arm. Compared to the thermometry-only control arm, the adjusted hazard ratio (aHR) of DFU in the thermometry + mHealth intervention arm adjusted by site was 2.12 (95% CI 0.96 – 4.68), and 1.44 (95% CI 0.65 – 3.22) adjusted by site and previous foot ulceration ( Table 2). The incidence of DFU in participants with previous foot ulceration was 23.7% (9/38) in the control arm and 30.8% (16/52) in the intervention arm, whereas in the participants without previous foot ulceration, incidence was 0% (0/38) in the control arm and 7.7% (2/26) in the intervention arm. Four participants did not have information related to their previous foot ulceration status (three from the control arm and one from the intervention arm).

**Table 2.** DFU incidence and effect of the intervention on primary and secondary outcomes.

	Incidence		Effect estimates *	
	Control arm	Intervention arm		
	n/N (%)	n/N (%)	HR (95% CI)	OR (95% CI)
**Primary outcome: DFU**				
Overall population	9/79 (11.4)	19/79 (24.1)		
Adjusted by site			2.12 (0.96 – 4.68)	--
Adjusted by previous foot ulceration			1.47 (0.66 – 3.30)	--
Adjusted by site and previous foot ulceration			1.44 (0.65 – 3.22)	--
**Secondary outcomes**				
≥80% daily temperature measurements				
Crude	54/59 (91.5%)	49/59 (83.1%)		0.45 (0.15 – 1.42)
Adjusted by site				0.46 (0.15 – 1.43)
Adjusted by site and previous foot ulceration				0.43 (0.13 – 1.40)
Reduction of ≥1% of glycosylated hemoglobin				
Crude	20/58 (34.5%)	14/50 (28.0%)		0.74 (0.33 – 1.68)
Adjusted by site				0.73 (0.32 – 1.67)
Adjusted by site and previous foot ulceration				0.64 (0.28 – 1.51)

* HRs were calculated among the 169 participants that had at least one follow-up evaluation during the 18-month study period. ORs were calculated among the 158 participants that finished the 18-months follow-up and had complete data to analysis. All effect estimates were calculated using the thermometry-only arm as the reference group.

DFU, diabetic foot ulcer; HR, hazard ratio; CI, confidence interval, OR, odds ratio.

### Secondary outcomes

The frequency of ≥80% of adherence to daily temperature measurement was 87.2% (103/118) among the study participants that returned the logbook. There was no evidence of a difference between study arms in the secondary outcomes of adherence to daily temperature measurements or reduction of HbA1c ( Table 2). Also, we found that 41% of the participants recorded an alarm sign in their logbooks. Additionally, 67% of the participants that presented an ulcer also reported an alarm sign in their logbook.

### Sub-group analyses in intervention vs. control arms

No effects of the intervention were found according to *a priori* pre-defined sub-groups. Among participants that did not have a caregiver (n=96), the aHR of developing a DFU was 3.34 (95% CI 0.94 – 11.92), adjusted by site and previous ulcer. Other results for sub-group analyses are shown in Table 3.

**Table 3.** DFU incidence and effect of the intervention by a *priori* defined sub-groups.

	DFU incidence		Effect estimate *
	Control arm	Intervention arm	
	n/N (%)	n/N (%)	HR (95% CI)
Among those who had a caregiver			
Crude	6/31 (19.4)	7/31 (22.6)	1.11 (0.37 – 3.32)
Adjusted by site and previous foot ulceration			0.43 (0.13 – 1.48)
Among those who did not had a caregiver			
Crude	3/48 (6.3)	12/48 (25.0)	**4.13 (1.16 – 14.63)**
Adjusted by site and previous foot ulceration			3.34 (0.94 – 11.92)

* HRs were calculated among the 169 participants that had at least one follow-up evaluation during the 18-month study period. All effect estimates were calculated using the thermometry-only arm as the reference group

DFU, diabetic foot ulcer; CI, confidence interval; HR, hazard ratio.

**Table 4.** Process evaluation of the use of thermometer at the 18-month follow-up.

Characteristics	N=102 n (%)
**TempStat™ Use: step by step**	
Use of TempStat™ in an illuminated area	8 (7.8%)
Use of TempStat™ immediately after wake up	100 (97.1%)
Use of the TemStat™ without socks and with warm feet	34 (33.0%)
Use of the TempStat™ seated in a chair, with the feet on the device and with the hands in the knees applying a little pressure	46 (44.7%)
Stay with the feet on the TempStat™ during 60 seconds	68 (66.0%)
Correct alarm sign identification	84 (81.6%)
Daily registration in the logbook	54 (52.4%)
**Logbook use**	
Participants using their logbooks	93 (90.3%)
**Nursing consultation**	
Report of communication with the study nurse	69 (66.9%)
Reason of the communication	
Consultation about TempStat™ use	1 (1.5%)
Alarm sign detection	5 (7.3%)
Schedule consultation	63 (91.2)
You consider that the nurse solved effectively your doubts or problems	66 (97.9%)

**Table 5.** Process evaluation of the mHealth strategy at the 18-month follow-up.

Characteristics	N=39 n (%)
**Messages**	
Always read the messages	27 (69.2%)
The messages help you a lot to improve your foot care	29 (74.4%)
The messages help you a lot to remember to use the device	27 (69.2%)
**Understanding of the messages**	
Daily thermometer usage	37 (94.9%)
Use of the TempStat™ during the morning	39 (100.0%)
Correct identification of alarm sign	30 (76.9%)
Correct actions if an alarm sign was detected	30 (76.9%)
Use of warm water to wash your feet	32 (82.1)
Avoid utilization of tight shoes	39 (100%)

### Sub-group analysis within the intervention arm

Participants were arranged according to the recipient of the mHealth reminders; the participants themselves (45/86) or the caregiver (41/86). We found no evidence of a difference in DFU incidence between these two groups in crude (HR 1.09, 95% CI 0.44 – 2.70), and adjusted analyses (aHR 1.72, 95% CI 0.65 – 4.54, adjusted by site and previous ulcer).

### Process evaluation indicators

Some process evaluation indicators for TempStat™ use and understanding of the messages are shown in Table 4 and Table 5. This data was obtained at the 18-month follow-up visit ^29, 30^.

***Dose of the mHealth component.*** The total number of messages to be sent to the patients in the intervention group during the study period was intended to be 86 text messages and 76 voice messages. The automated software system sent <50% of the intended SMS and voice messages to 1/86 (1.2%) of the participants, between 50–75% of the messages to 18/86 (20.9%) of the participants, and ≥75% of the messages to 67/86 (77.9%) of the participants. In contrast, text and voice messages received by the participants was <50% for 42/86 (48.8%) of the participants, and between 50% and 75% for 44/86 (51.2%) of the participants.

***Preferences of SMS or voice messages.*** Among 101 interviewees (one participant did not answer), 42.6% preferred text messaging, whereas 57.4% preferred voice messages. Those who preferred voice messaging over SMS generally had that preference because they had difficulty reading text messages on the cell phone screen. Other participants with this preference mentioned that they have quicker access to the information with a voice message. Those who preferred SMS for reminders cited the fact that SMS can be read at their convenience. Some mentioned that they prefer SMS because they don’t want to have to listen for phone calls and/or pay attention to their phone at certain times.

Some participants commented that regardless of the reminder system (SMS or voice messaging), it was necessary to receive help from other people to read or listen to the messages. Their children were most commonly cited as the people to whom the participants would turn for help.

***Use of TempStat™.*** Some participants mentioned that they had some periods during which they did not use the device. Among the reasons provided were that the device had technical problems or because they did not have the logbook to record their measurements.

***Suggestions.*** Among the suggestions to improve the device and its use, technical comments were the most common. Participants mentioned that they preferred a smaller size and lighter weight device. Furthermore, of the 8% of participants that had to replace the TempStat™ because of technical problems, some mentioned that an improved design could increase the lifetime of the device. Additionally, participants found the reinforcement of the logbook and device utilization by the nurses to be very important, and some commented that more frequent communication with the nurse could improve compliance with device use.

## Discussion

### Main findings

This study was designed to compare the 18-month incidence of DFU between those receiving thermometry + mHealth reminders versus thermometry-only. The uptake of the thermometry was high in this study, nearly 90% of the participants who returned the logbook had achieved ≥80% of the daily feet temperature measurements. At baseline, we unexpectedly found a higher prevalence of previous foot ulceration in the intervention arm, and the incidence of DFU was higher in this arm. In our study, conducted in a low-income setting, the addition of mHealth was not effective in reducing foot ulceration or increasing adherence to thermometry after 18 months of follow-up. However, these results need to be interpreted with caution as the expected incidence rates of DFU used in our sample size calculations were not met and there was a higher rate of previous DFU in the intervention group. 

### Comparison to previous studies

In our cohort, according to the process evaluation results, adherence to temperature measurement was good, procedures about how to use the TempStat™ were regular (some steps have less than 50% of correct answers) and correct alarm sign detection was good (81%). One previous study using thermometry found that 80% of participants who developed an ulcer did not comply with 50% of the temperature assessments, in contrast with the group that did not develop an ulcer, where 92% of participants recorded their foot temperatures at least half the time ^7^.

Also, in our results, 41% (44/108) of the study participants recorded alarm signs for two consecutive days in their logbooks, and we only have data from 9/44 (20%) that had a record of reporting an alarm sign to the study nurse. These figures do not consider those with alarm signs that did not seek nurse support or those who did report to the nurse but their report was not recorded.

The low rate of ulceration occurrence in our study could be potentially explained by two factors. First, that the participants did follow the instructions to reduce physical activity when observing alarm signs, even when they were not for two consecutive days or if they did not seek or receive the feedback of the study nurse. This is because the recommendations about reducing foot pressure and physical activity were given at the beginning of the study (videos) and they were also printed in their logbooks. Secondly, it is possible that the frequent assessment of the participant by the study nurse, every two months, may have played a role among study participants, including the control group. These two could have contributed to the lack of effect of the mHealth component in reducing foot ulceration.

Health interventions using SMS for diabetes management have been found to be useful for improving self-efficacy and social support ^11^, as well as clinical diabetes-related outcomes ^12^. However, most of the mHealth studies were conducted in high-income countries, with a young population and with outcomes related to HbA1c measurements or questionnaires, without evaluating patient important outcomes like mortality, complications or quality of life. Despite the perceived benefit of mHealth in the elderly population ^31^, very few studies with this population have been conducted in LMICs. Our automatic system delivered >75% of the messages to two-thirds of the participants only and it did not have a human support component, factors that may have affected the effective engagement with the mHealth intervention ^32, 33^. For example, a previous study using tailored motivational phone calls followed by SMS in people with pre-hypertension found a larger effect on bodyweight and waist circumference reduction in participants that received ≥75% of the calls ^34^. Additionally, our system was automatic and did not allow direct bilateral communication. In a previous qualitative study from Canada, conducted to explore the views of patients in using mHealth to monitor and prevent DFU ^35^, patients expressed interest in a two-way communication system to facilitate sharing of medical data, scheduling appointments and using of alerts to get access to medical attention. Also, a recent publication, evaluating 17 systematic reviews of mHealth intervention studies in diabetes and obesity ^36^, showed that fewer than half of the studies included in 2 reviews (out of 7 systematic reviews that covered the topic) improved diabetes management practices or medication adherence ^37, 38^, and recommend the use of valid measures for outcomes and rigorous study designs to improve their quality. Finally, compared to previous mHealth studies where the focus has been on laboratory parameters or questionnaires ^39, 40^, we measured the impact of mHealth on DFU, an outcome of patient importance.

### Limitations and strengths

Our study has some limitations. At baseline, participants assigned to the intervention arm were at higher risk of DFU, and the ulceration rate observed in the study was lower than expected. Together these reduced the accrual of sufficient DFU events despite extending the study from 12 to 18 months. Also, we did not collect information about the duration since the most recent wound healed. Recent research suggests ~10% of wounds recur within a month and 40% within a year of entering diabetic foot remission. Also, adherence to foot temperature measurements was self-reported, and the adherence to the recommendations of the reduction of physical activity was not recorded, not being able to characterise certain behaviours of direct relevance to our DFU outcome. Our sample size calculations, which were made with an absolute change in DFU of 21% between the intervention and the control arm (9% vs 30%), using data derived from studies in high income countries, were different from the incidence of DFU observed in our trial. Hence, it is possible that our study was underpowered to detect the expected effects. Finally, it is possible that those who did not return the logbook (~30%), where alarm signs were to be recorded, may be less conscientious about foot temperature measurements and thus may have had lower rates of adherence to the thermometry.

The study also has some strengths; namely, it is a practical and pragmatic trial, well protected from bias, measuring an outcome of importance to patients and inclusive of low-income patients over 60 years-old attending public hospitals in a middle-income country.

### Relevance to public health

The experience of introducing a device to engage with self-care behaviors for the prevention of DFU in a LMIC setting showed good adherence rates in both study arms, nearly reaching 90%, signaling that mHealth had little room to further exert an impact. Future studies could pre-select participants with low adherence and explore if mHealth appears as a good supplement to prevent DFU.

Maintaining such DFU prevention efforts in routine clinical settings may be difficult to sustain, yet this study demonstrates that adequate promotion of foot care can be achieved.

## Conclusions

In this randomized trial, conducted in a LMIC setting, the uptake of the foot thermometry for the prevention of foot ulcers was 87% in the intervention and control groups, and the addition of mHealth was not effective in reducing foot ulceration or increasing adherence to thermometry after 18 months of follow-up. However, these results need to be interpreted with caution as the expected rates of DFU used in our sample size calculations were not met and there was a higher rate of previous DFU in the intervention group.

## Data availability

### Underlying data

Figshare: Database main analysis. https://doi.org/10.6084/m9.figshare.11310827.v2 ^28^

Figshare: Dictionary main database. https://doi.org/10.6084/m9.figshare.11478003.v1 ^41^

Figshare: Database process evaluation. https://doi.org/10.6084/m9.figshare.11317601.v2 ^29^

Figshare: Dictionary process evaluation. https://doi.org/10.6084/m9.figshare.11477985.v1 ^42^

Figshare: Transcripts. https://doi.org/10.6084/m9.figshare.11310740.v1 ^30^

*Extended data*

Figshare: Validation of mHealth messages. https://doi.org/10.6084/m9.figshare.11310620.v1 ^20^

Figshare: Messages used in the mHealth component. https://doi.org/10.6084/m9.figshare.11310665.v1 ^23^

Figshare: Questionnaires. https://doi.org/10.6084/m9.figshare.11310548.v2 ^25^

*Reporting guidelines*

Figshare: CONSORT checklist. https://doi.org/10.6084/m9.figshare.11310512.v1 ^43^

Data are available under the terms of the Creative Commons Attribution 4.0 International license (CC-BY 4.0).

## Software availability

Source code available from: https://github.com/dgnest/foottrial

Archived source code at time of publication: https://doi.org/10.5281/zenodo.3628824 ^24^

License: Apache 2.0
