# Supplementary material for: Foot thermometry with mHeath-based supplementation to prevent diabetic foot ulcers: A randomized controlled trial
Source: Wellcome Open Res. 2020 Aug 28;5:23. Originally published 2020 Feb 10. [Version 2] doi: 10.12688/wellcomeopenres.15531.2 (PMC7463300; doi:10.12688/wellcomeopenres.15531.2)
Supplement: Peer review history

# Peer review: Reviewer response for version 2

**DOI:** 10.21956/wellcomeopenres.17701.r40205

**Published:** 2020-09-04

**Authors:** Peter Mertens

**Affiliations:** 1 Clinic of Nephrology, Hypertension, Diabetes and Endocrinology, Otto-von-Guericke University Magdeburg, Magdeburg, Germany

I have no further comments for improvement. The authors have adequately addressed my suggestions.

Is the work clearly and accurately presented and does it cite the current literature?

Yes

If applicable, is the statistical analysis and its interpretation appropriate?

Yes

Are all the source data underlying the results available to ensure full reproducibility?

Yes

Is the study design appropriate and is the work technically sound?

Yes

Are the conclusions drawn adequately supported by the results?

Partly

Are sufficient details of methods and analysis provided to allow replication by others?

Yes

Reviewer Expertise:

Diabetes and secondary diseases, complications, endocrinology, nephrology.

I confirm that I have read this submission and believe that I have an appropriate level of expertise to confirm that it is of an acceptable scientific standard.

# Peer review: Reviewer response for version 2

**DOI:** 10.21956/wellcomeopenres.17701.r40207

**Published:** 2020-09-01

**Authors:** Jaap J. van Netten

**Affiliations:** 1 Department of Rehabilitation, Amsterdam Movement Sciences, Amsterdam UMC, University of Amsterdam, Amsterdam, The Netherlands

I'm happy with the changes made following peer review.

Is the work clearly and accurately presented and does it cite the current literature?

Yes

If applicable, is the statistical analysis and its interpretation appropriate?

Partly

Are all the source data underlying the results available to ensure full reproducibility?

Yes

Is the study design appropriate and is the work technically sound?

Yes

Are the conclusions drawn adequately supported by the results?

Yes

Are sufficient details of methods and analysis provided to allow replication by others?

Yes

Reviewer Expertise:

Diabetic foot disease, biomechanics, human movement sciences.

I confirm that I have read this submission and believe that I have an appropriate level of expertise to confirm that it is of an acceptable scientific standard.

# Peer review: Reviewer response for version 2

**DOI:** 10.21956/wellcomeopenres.17701.r40206

**Published:** 2020-09-01

**Authors:** Javier Ena

**Affiliations:** 1 Hospital Marina Baixa, Alicante, Spain

Is the work clearly and accurately presented and does it cite the current literature?

Yes

If applicable, is the statistical analysis and its interpretation appropriate?

Yes

Are all the source data underlying the results available to ensure full reproducibility?

Yes

Is the study design appropriate and is the work technically sound?

Yes

Are the conclusions drawn adequately supported by the results?

Yes

Are sufficient details of methods and analysis provided to allow replication by others?

Yes

Reviewer Expertise:

Diabetes complications

I confirm that I have read this submission and believe that I have an appropriate level of expertise to confirm that it is of an acceptable scientific standard.

# Peer review: Reviewer response for version 1

**DOI:** 10.21956/wellcomeopenres.17001.r38648

**Published:** 2020-06-15

**Authors:** Peter Mertens

**Affiliations:** 1 Clinic of Nephrology, Hypertension, Diabetes and Endocrinology, Otto-von-Guericke University Magdeburg, Magdeburg, Germany

The manuscript submitted by Lazo-Porras *et al*. entitled “Foot thermometry with mHealth-based supplementation to prevent diabetic foot ulcers: A randomized controlled trial” is a well performed clinical study with published study protocol and enormous effort to ensure proper sampling of data.

 The main finding of the study is reported as a negative outcome finding: mHealth does not add to the adherence of the patients at risk for diabetic foot ulceration to perform daily thermometry. There may be numerous interpretations and reasons for this negative result, which at first sight surprises and is counterintuitive to the general field.

 As referee I ask myself whether this is unexpected or whether there is an outlying explanation. The study protocol states: “Periodic assessments of the participants involving a general checkup and lower extremity evaluation was conducted every two months by the nurse evaluator.” Thus every participant was seen at least every second month by the nurse practitioner and the conversations within this assessment may be more important than the messages (either voice or text messages). What was the duration of the consultations? Was it standardized? It is my impression that these contacts may have skewed the results markedly and these effects have not been tested, as far as I understand the study.

 Another important aspect may be that the alarms have not been analyzed in their consequences. Have there been additional contacts to the study centre? Have these been recorded? How may a bias been excluded.

 In addition there is an unfortunate bias due to more DFU in the intervention group which makes it difficult to draw solid conclusions.

 The statements regarding the effectiveness of mHealth should be weakened markedly given the limitations and shortcomings of the study. These should therefore be stated in the respective chapters (abstract, discussion,conclusions).

Is the work clearly and accurately presented and does it cite the current literature?

Yes

If applicable, is the statistical analysis and its interpretation appropriate?

Yes

Are all the source data underlying the results available to ensure full reproducibility?

Yes

Is the study design appropriate and is the work technically sound?

Yes

Are the conclusions drawn adequately supported by the results?

Partly

Are sufficient details of methods and analysis provided to allow replication by others?

Yes

Reviewer Expertise:

Diabetes and secondary diseases, complications, endocrinology, nephrology.

I confirm that I have read this submission and believe that I have an appropriate level of expertise to confirm that it is of an acceptable scientific standard, however I have significant reservations, as outlined above.

# Response

**Published:** 2020-07-01

**Authors:** Maria Lazo-Porras

**Affiliations:** Universidad Peruana Cayetano Heredia, Peru

1. The manuscript submitted by Lazo-Porras et al. entitled “Foot thermometry with mHealth-based supplementation to prevent diabetic foot ulcers: A randomized controlled trial” is a well performed clinical study with published study protocol and enormous effort to ensure proper sampling of data. 

**Response: We very much appreciate your comment. Thank you.**

2. The main finding of the study is reported as a negative outcome finding: mHealth does not add to the adherence of the patients at risk for diabetic foot ulceration to perform daily thermometry. There may be numerous interpretations and reasons for this negative result, which at first sight surprises and is counterintuitive to the general field. 

As referee I ask myself whether this is unexpected or whether there is an outlying explanation. The study protocol states: “Periodic assessments of the participants involving a general checkup and lower extremity evaluation was conducted every two months by the nurse evaluator.” Thus every participant was seen at least every second month by the nurse practitioner and the conversations within this assessment may be more important than the messages (either voice or text messages). What was the duration of the consultations? Was it standardized? It is my impression that these contacts may have skewed the results markedly and these effects have not been tested, as far as I understand the study.

**Response: The reviewer raises an important point related to the frequency of contacts between the study participants and the health system, which may help understanding the negative effects of our intervention. Prior to the study, we do not have any information about the participant’s frequency of contact with the health system. In principle, as part of the existing usual care, patients may be asked to visit their doctor every three months and whether or not they meet that criteria may have been affected by a variety of reasons, e.g. distance, transportation, availability of an appointment, costs, etc.  So, yes, we concur with the observation that our study promoted more frequent visits to the health system than usual care, and this may have had a role in the prevention of foot ulceration.**

**Also, we did not collect the duration of the follow-up visit but the assessment included a questionnaire about medication, use of shoes and/or insoles, presence of a caregiver, blood pressure measurement and foot evaluation. These interactions could have impact the study findings skewed the results markedly.** **We added the following in the manuscript:**

***“The low rate of ulceration occurrence in our study could be potentially explained by two factors. First, that the participants did follow the instructions to reduce physical activity when observing alarm signs, even when they were not for two consecutive days or if they did not seek or receive the feedback of the study nurse. This is because the recommendations about reducing foot pressure and physical activity were given at the beginning of the study (videos) and they were also printed in their logbooks. Secondly, it is possible that the frequent assessment of the participant by the study nurse, every two months, may have played a role among study participants, including the control group.  These two could have contributed to the lack of effect of the mHealth component in reducing foot ulceration.”***

3. Another important aspect may be that the alarms have not been analyzed in their consequences. Have there been additional contacts to the study centre? Have these been recorded? How may a bias been excluded? 

**Response: Information about the alarms reported to the study nurse was low. However, we do not have reports about additional contacts with the study sites. What we know is that some participants maintained their usual care appointments with their endocrinologists.**

4. In addition there is an unfortunate bias due to more DFU in the intervention group which makes it difficult to draw solid conclusions. 

**Response: This is true, and we concur. For that reason, we already discuss this as a limitation of the study.**

5. The statements regarding the effectiveness of mHealth should be weakened markedly given the limitations and shortcomings of the study. These should therefore be stated in the respective chapters (abstract, discussion, conclusions). 

**Response: Yes. This is correct. We have refined our conclusions (see Reviewer 1, response #2).**

# Peer review: Reviewer response for version 1

**DOI:** 10.21956/wellcomeopenres.17001.r38649

**Published:** 2020-05-26

**Authors:** Javier Ena

**Affiliations:** 1 Hospital Marina Baixa, Alicante, Spain

This is a well-conducted randomized clinical trial including 172 patients at risk of DFU. Randomization technique was well carried out, but unfortunately, there was a greater risk for DFU in the intervention group due to a greater proportion of previous DFU. The intervention was well explained. However, the authors did not assess patients' adherence to recommendations to reduce daily exercise.

 Finally, the clinical trial did not show the advantage of using foot thermometry and mHealth supplementation to reduce DFU.

 A big caveat of the study is the sample size. According to my estimates, the number of patients included in the clinical trial was too small to show a possible benefit of the intervention. Taking into account a risk of DFU in the control group of 24%, a reduction of 50% in the risk of DFU in the intervention group with a power of 80% and a two-sided alfa error of 5%, the sample size called for 159 patients per arm (Fleiss estimation). 

 In summary, it is not clear whether foot thermometry with mHealth-based supplementation is useful or not to prevent DFU.

Is the work clearly and accurately presented and does it cite the current literature?

Yes

If applicable, is the statistical analysis and its interpretation appropriate?

Yes

Are all the source data underlying the results available to ensure full reproducibility?

Yes

Is the study design appropriate and is the work technically sound?

Yes

Are the conclusions drawn adequately supported by the results?

Yes

Are sufficient details of methods and analysis provided to allow replication by others?

Yes

Reviewer Expertise:

Diabetes complications

I confirm that I have read this submission and believe that I have an appropriate level of expertise to confirm that it is of an acceptable scientific standard, however I have significant reservations, as outlined above.

# Response

**Published:** 2020-07-01

**Authors:** Maria Lazo-Porras

**Affiliations:** Universidad Peruana Cayetano Heredia, Peru

1. This is a well-conducted randomized clinical trial including 172 patients at risk of DFU. Randomization technique was well carried out, but unfortunately, there was a greater risk for DFU in the intervention group due to a greater proportion of previous DFU. The intervention was well explained. However, the authors did not assess patients' adherence to recommendations to reduce daily exercise.

Finally, the clinical trial did not show the advantage of using foot thermometry and mHealth supplementation to reduce DFU. 

**Response: Thank you for your feedback. Yes, we did not measure adherence to the recommendations of the reduction of daily physical activity and for that reason we are adding that as a limitation:** 

***“Also, adherence to measurements was self-reported, and the adherence to the recommendations of the reduction of physical activity was not recorded, not being able to characterise certain behaviours of direct relevance to our DFU outcome."***

2. A big caveat of the study is the sample size. According to my estimates, the number of patients included in the clinical trial was too small to show a possible benefit of the intervention. Taking into account a risk of DFU in the control group of 24%, a reduction of 50% in the risk of DFU in the intervention group with a power of 80% and a two-sided alfa error of 5%, the sample size called for 159 patients per arm (Fleiss estimation).  

**Response: Thank you for your comment. We estimated the sample size** **with an absolute change of 21% between the intervention arm and the control arm (9% vs 30%). However, these data came from previous results in high income countries and differs from the incidence of diabetic foot ulcer results observed in our trial.  We added the following information in the manuscript.**

***Our sample size calculations, which were made with an absolute change of 21% between the intervention and the control arm (9% vs 30%), using data derived from studies in high income countries, were different from the incidence of DFU results observed in our trial. Hence, it is possible that our study was underpowered to detect the expected effects”***

3. In summary, it is not clear whether foot thermometry with mHealth-based supplementation is useful or not to prevent DFU.

**Response: This is correct. We have refined our conclusions (see Reviewer 1, response #2).**

# Peer review: Reviewer response for version 1

**DOI:** 10.21956/wellcomeopenres.17001.r38384

**Published:** 2020-04-17

**Authors:** Jaap J. van Netten

**Affiliations:** 1 Department of Rehabilitation, Amsterdam Movement Sciences, Amsterdam UMC, University of Amsterdam, Amsterdam, The Netherlands

This is a well-performed RCT, with clear protocol published in advance, and following general rules for RCTs. The manuscript is easy to read, has clear description of results, and adds to the body of knowledge in the field of diabetic foot disease.

 I have some comments to improve understanding and interpretation of the findings, and I think the authors can be more clear in describing the negative results of the intervention. I will discuss these per section.

Abstract:

 The conclusion from the abstract is not a conclusion and not informative. This should be something along the lines of the primary outcome and primary research question, such as “mHealth is not effective in reducing foot ulceration or increasing adherence to thermometry”.

Introduction:

 It is stated that “3 trials have been performed on thermometry”. The fourth trial that is available on this topic should also be mentioned here (Skafjeld *et al.*) ^1^, and perhaps some of the meta-analyses as well (e.g. Crawford *et al*, 2019) ^2^.

 Importance of interventions to improve adherence to diabetic foot self-care is also described in various other papers, such as the IWGDF systematic review on preventative interventions and the review on modifiable interventions (Van Netten *et al.* 2020a and 2020b) ^3^^,^^4^, or papers on adherence in relation to diabetic foot disease (e.g. Price 2016) ^5^. The introduction would improve in depth if importance of adhering to these aspects of diabetic foot self-care would also be included, rather than focusing only on thermometry.

Methods:

 It is stated that the previous trials only included IWGDF3, but that is not correct. At least one temperature trial (Lavery *et al.*) ^6^ included also IWGDF2 patients.

 How was education at baseline delivered? Verbal, written, pictures, videos?

 The TempStat is used, but no information is provided in the methods about the validity and reliability of this instrument.

 Was there any way to assess if alerts appeared, other than the logbook? Did authors test this instrument in advance?

 From the sentence: “In the intervention arm, additional to the TempStat™ participants received the mHealth component (two reminder messages and six foot-care promotion messages) during the 18-month study period via both SMS and voice messaging.” it reads as if these messages were only sent 8 times throughout the entire period. However, in a subsequent paragraph it’s clear they were sent weekly. Please rephrase this sentence to make sure it reflects what happened.

 It is stated that alarms were not analysed “because of their low frequency.” However, with 28 ulcers developed, one would expect a fair amount of warnings (if the skin indeed heats up before it breaks down). Can the authors explain why alarms were of such low frequency?

 Is it correct that the blinded evaluator was evaluating the ulcers in real-life, not via photos? i.e.: patients could tell which arm they were in, albeit inadvertently?

 Randomization via envelopes is not considered independent anymore, since these can be (easily) manipulated. Please describe what was done to prevent this.

Results:

 Flowchart: add “n=” in the last box (e.g. n=59), to avoid that readers may think that this is an outcome (because HbA1c of 50 is a potential finding).

 Flowchart: are there any reasons for lost to follow-up?

 Baseline: any information on comorbidities, such as cardiac disease?

 Baseline: can authors provide some additional info on foot deformities? What sort of deformities were found, how were these assessed?

 I miss information on footwear and other preventative self-care of the patients throughout the trial.

 Why were no multivariate analyses done?

Discussion

 Main findings: the actual main finding is somewhat obscured in this paragraph. It is described as “At baseline, we unexpectedly found a higher prevalence of previous foot ulceration in the intervention arm, and the incidence of DFU was higher in this arm.”. However, these are two separate findings. The current presentation suggests causality, but that’s not the case. Because it is clear in the results that even a subanalysis in patients with a previous DFU shows higher incidence during the study in the intervention group (23% vs 30%). Authors should be crystal clear in their main finding here, see also my comment concerning the abstract. The finding is simple: mHealth does not help.

 In the “comparison to previous studies”, the actual findings are not discussed. Part of this section is now a simple repetition of the introduction, the other parts fail to acknowledge the negative findings of the current study. Why does mHealth appear to be beneficial in other studies, but not in this study? That is what should be reflected on.

 I miss any discussion about the comment that alarms were not evaluated because of their low frequency. That implies that thermometry is not useful in ulcer prevention. This should be discussed in depth.

 Ulcer incidence in patients with a previous ulcer is 27.8%, which is somewhat lower than other studies. Can this be attributed to the thermometer? And if so, how, if the thermometer never gives an alarm? Or is this because usual care is really good?

 I miss discussion of the reliability and validity of the instrument, or the lack of knowledge thereof.

 In the limitation section, I miss acknowledging limitations in randomization (done with envelopes) and blinding (outcome assessor could be unblinded by patients). See comments in method section.

Conclusions:

 Authors have a more clear conclusion in this section (first sentence). That is what should be used in the abstract and discussion.

Is the work clearly and accurately presented and does it cite the current literature?

Yes

If applicable, is the statistical analysis and its interpretation appropriate?

Partly

Are all the source data underlying the results available to ensure full reproducibility?

Yes

Is the study design appropriate and is the work technically sound?

Yes

Are the conclusions drawn adequately supported by the results?

Yes

Are sufficient details of methods and analysis provided to allow replication by others?

Yes

Reviewer Expertise:

Diabetic foot disease, biomechanics, human movement sciences.

I confirm that I have read this submission and believe that I have an appropriate level of expertise to confirm that it is of an acceptable scientific standard, however I have significant reservations, as outlined above.

## References

[1] : A pilot study testing the feasibility of skin temperature monitoring to reduce recurrent foot ulcers in patients with diabetes--a randomized controlled trial. *BMC Endocr Disord*.2015;15: 10.1186/s12902-015-0054-x 55 10.1186/s12902-015-0054-x 26452544PMC4600271

[2] : Preventing foot ulceration in diabetes: systematic review and meta-analyses of RCT data. *Diabetologia*.63(1) : 10.1007/s00125-019-05020-7 49-64 10.1007/s00125-019-05020-7 31773194PMC6890632

[3] : Treatment of modifiable risk factors for foot ulceration in persons with diabetes: a systematic review. *Diabetes Metab Res Rev*.36 Suppl 1: 10.1002/dmrr.3271 e3271 10.1002/dmrr.3271 31957306

[4] : Prevention of foot ulcers in the at-risk patient with diabetes: a systematic review. *Diabetes Metab Res Rev*.36 Suppl 1: 10.1002/dmrr.3270 e3270 10.1002/dmrr.3270 31957213

[5] : How can we improve adherence?. *Diabetes Metab Res Rev*.2016;32 Suppl 1: 10.1002/dmrr.2744 201-5 10.1002/dmrr.2744 26453542

[6] : Home monitoring of foot skin temperatures to prevent ulceration. *Diabetes Care*.2004;27(11) : 10.2337/diacare.27.11.2642 2642-7 10.2337/diacare.27.11.2642 15504999

# Response

**Published:** 2020-07-01

**Authors:** Maria Lazo-Porras

**Affiliations:** Universidad Peruana Cayetano Heredia, Peru

1. This is a well-performed RCT, with clear protocol published in advance, and following general rules for RCTs. The manuscript is easy to read, has clear description of results, and adds to the body of knowledge in the field of diabetic foot disease. 

**Response: Thank you for your positive feedback.**

2. I have some comments to improve understanding and interpretation of the findings, and I think the authors can be more clear in describing the negative results of the intervention. I will discuss these per section. 

Abstract:

The conclusion from the abstract is not a conclusion and not informative. This should be something along the lines of the primary outcome and primary research question, such as “mHealth is not effective in reducing foot ulceration or increasing adherence to thermometry”.

**Response: Thank you for your suggestion, we have added a paragraph based on your recommendation but also including suggestions of the other two reviewers (see reviewer 2 comments #2 and #3, and reviewer 3 comments #2, #5 and #6). The Abstract’s conclusion now reads:**

***“In our study, conducted in a low-income setting, the addition of mHealth to foot thermometry was not effective in reducing foot ulceration. Importantly, there was a higher rate of previous DFU in the intervention group, the adherence to thermometry was high, and the expected rates of DFU used in our sample size calculations  were not met.”***

3. Introduction:

It is stated that “3 trials have been performed on thermometry”. The fourth trial that is available on this topic should also be mentioned here (Skafjeld *et al.*) ^1^, and perhaps some of the meta-analyses as well (e.g. Crawford *et al*, 2019) ^2^.

**Response: We agree with your comment and we have added these articles in our introduction section.**

***“Three previous clinical trials and one systematic review have found that the use of thermometry reduced DFU incidence four- to ten-fold among individuals with diabetes at high-risk of developing a DFU*** ***^1^*** ***.*** ***Additionally, one study found that the addition of counselling to promote self-monitoring of skin temperature to standard care is feasible*** ***^2^*** ***.”***

4. Importance of interventions to improve adherence to diabetic foot self-care is also described in various other papers, such as the IWGDF systematic review on preventative interventions and the review on modifiable interventions (Van Netten *et al.* 2020a and 2020b) ^3^ ^,^ ^4^, or papers on adherence in relation to diabetic foot disease (e.g. Price 2016) ^5^. The introduction would improve in depth if importance of adhering to these aspects of diabetic foot self-care would also be included, rather than focusing only on thermometry.

**Response: Thank you for your suggestion. We have expanded our introduction to include evidence about the importance of foot care.**

***“Other approaches that could prevent foot ulcers include patient’s foot self-care behaviour, annual foot evaluations, knowledge about diabetic foot in health care workers, and therapeutic footwear ^3^. Also, in order to prevent recurrent ulcers, it is important to consider the integration or combination of these approaches ^4^."***

5. Methods:

It is stated that the previous trials only included IWGDF3, but that is not correct. At least one temperature trial (Lavery *et al.*) ^6^ included also IWGDF2 patients.

**Response: Yes, that it is true. We have changed the information accordingly.** 

***“Our eligibility criteria used IWGDF categories and included people with diabetes at risk of ulceration group 2 and 3. In so doing, rather than focusing only on those at the highest risk for ulceration (IWGDF group 3) we wanted to pursue a pragmatic approach for the prevention of DFU among people with diabetes, thus including also those participants from the IWGDF group 2 category. All previous studies included mostly participants from IWGDF group 3, and only one clinical trial included group 2 patients.”***

6. How was education at baseline delivered? Verbal, written, pictures, videos?

**Response: We used videos to educate participants about foot care. We have clarified this information in the article.** 

***“At the initiation visit, all participants received education about foot care, i.e. etiology and risk factors for the development of neuropathy and ulcers, as well as recommendations for foot care practices and early signs of ulceration; and instructions for the use of the TempStat™ device (see Extended data). This foot care education was done through three videos that were validated by physicians and patients with type 2 diabetes mellitus. The first two videos lasted 8 and 6 minutes and they were related to foot care, whereas the third video lasted 6 minutes and presented the instructions on the use of the TempStat™ device. The three videos were in Spanish and were showed once at the initiation visit.”***

7. The TempStat is used, but no information is provided in the methods about the validity and reliability of this instrument. 

**Response:  We included some of this information in our protocol, already published** **^5^** **. But we also think that it is a good suggestion and we decided to include this in the article.**

***“Frykberg et al. ^6^ showed that TempStat™ can detect alarm signs, represented by a yellow color change, and the results positively correlate to temperature findings of infrared thermometer, the gold standard of thermometry devices. Another study found that the device identified 74% of serious foot problems ^7 ^"***

8. Was there any way to assess if alerts appeared, other than the logbook? Did authors test this instrument in advance? 

**Response: We did not have another method to assess this during the study, other than using the logbook. Before embarking on this strategy, we piloted the TempStat *™* and logbook with 10 patients, for two weeks, to know if the participants understood the instructions of the video about how to use the TempStat *™* and if they recorded adequately their information in the logbook. We verified that they had understood the instructions and completed the logbook correctly. We know that this is not the best method because of the information being self-reported but, given the pragmatic nature of the study, it was the most feasible approach in the study setting where our study was carried out. During the conduction of the study, between 2015 and 2016, many patients did not yet have a smartphone or access to other technologies to ask them to send us an objective image of the TempStat *™*. We include this information as a limitation in the discussion.**

***“Also, adherence to measurements was self-reported, which potentially introduced bias.and the adherence to the recommendations of the reduction of physical activity was not recorded, not being able to characterise certain behaviours of direct relevance to our DFU outcome.”***

***“Finally, it is possible that those who did not return the logbook (~30%), where alarm signs were to be recorded, may be less conscientious about foot temperature measurements and thus may have had lower rates of adherence to the thermometry.”***

9. From the sentence: “In the intervention arm, additional to the TempStat™ participants received the mHealth component (two reminder messages and six foot-care promotion messages) during the 18-month study period via both SMS and voice messaging.” it reads as if these messages were only sent 8 times throughout the entire period. However, in a subsequent paragraph it’s clear they were sent weekly. Please rephrase this sentence to make sure it reflects what happened. 

**Response: Thank you for your comment. We have clarified this section because the messages were sent weekly.**

***“In the intervention arm, in addition to the TempStat™, participants received the mHealth component weekly (two reminder messages and six foot-care promotion messages each week) for the 18-month study period via both SMS and voice messaging.”***

10. It is stated that alarms were not analysed “because of their low frequency.” However, with 28 ulcers developed, one would expect a fair amount of warnings (if the skin indeed heats up before it breaks down). Can the authors explain why alarms were of such low frequency? 

**Response: The report of the alarms to the nurses was low, but records from the logbooks showed that 41% of the participants reported an alarm sign (we only considered an alarm sign if it was reported during 2 or more consecutive days). Additionally, 67% of the participants that presented an ulcer reported an alarm sign in the logbook.  We have now included the following information in the manuscript**

***“Also, we found that 41% of the participants recorded an alarm sign in their logbooks. Additionally, 67% of the participants that presented an ulcer also reported an alarm sign in their logbook.”***

11. Is it correct that the blinded evaluator was evaluating the ulcers in real-life, not via photos? i.e.: patients could tell which arm they were in, albeit inadvertently? 

**Response: The blinded evaluator saw the patient every two months, and if an ulcer was developed in this period the patient could also attend to an additional visit to the hospital to receive care and to be evaluated by the blinded evaluator. Participants were recommended not to mention their study arm (thermometry + mHealth vs. thermometry only). We did not receive any report of the blinded nurses related to a protocol deviation.**

12. Randomization via envelopes is not considered independent anymore, since these can be (easily) manipulated. Please describe what was done to prevent this. 

**Response: The procedure was as follow, the patient received a code in the hospital when it was recruited by the study nurse, then the study coordinator opened the envelop containing details of the random allocation of the participant to the intervention or the control group. So, it was not possible to the study nurse to know the allocation of the participant, and it was not possible to the study coordinator to evaluate the participant and give the code to him/her. We are providing more details in our manuscript:**

***“We conducted stratification using the hospital site as a single stratum and blocks of 6 to generate a random allocation sequence. Sealed envelopes with codes to randomize participants were used. An independent researcher prepared the envelopes, and the study nurses assigned the codes to each of the enrolled participants. Separately, the study coordinator was responsible for opening the envelopes and informing participants about their intervention or control allocation as per the random list. The nurse/independent evaluators were not aware of the patient's group allocation.”***

Results

13. Flowchart: add “n=” in the last box (e.g. n=59), to avoid that readers may think that this is an outcome (because HbA1c of 50 is a potential finding). 

**Response: Thank you for your suggestion, we added the “n=”**

14. Flowchart: are there any reasons for lost to follow-up? 

**Response: In our study, lost to follow-up were study participants where re-contact was not possible: some of them migrated back to their place of origin, and in other cases it was not possible to find the addresses provided, or the participant did not answer the phone calls. We have added these details in the manuscript:**

***“Reasons for lost to follow-up included migration back to the participant’s place of origin, wrong/incomplete addresses provided, or the participant did not answer the contact phone calls.”***

15. Baseline: any information on comorbidities, such as cardiac disease? 

**Response: Yes, that information is already provided in Table 1.**

16. Baseline: can authors provide some additional info on foot deformities? What sort of deformities were found, how were these assessed? 

**Response: The study nurses evaluated the deformities during the screening assessment, and we only considered 4 types of deformities: claw foot (40.5%), prominent metatarsal head (33.3%), Charcot foot (9.5%), and hammer toe (19.1%). This information is provided in the baseline characteristics section.**

17. I miss information on footwear and other preventative self-care of the patients throughout the trial. 

**Response: Less than 10% of the participants reported using orthopedic shoes or insoles. This information is available in Table 1.**

18. Why were no multivariate analyses done? 

**Response: Yes, we conducted a multivariate analyses adjusting by site and history of previous ulcer.**

Discussion

19. Main findings: the actual main finding is somewhat obscured in this paragraph. It is described as “At baseline, we unexpectedly found a higher prevalence of previous foot ulceration in the intervention arm, and the incidence of DFU was higher in this arm.” However, these are two separate findings. The current presentation suggests causality, but that’s not the case. Because it is clear in the results that even a subanalysis in patients with a previous DFU shows higher incidence during the study in the intervention group (23% vs 30%). Authors should be crystal clear in their main finding here, see also my comment concerning the abstract. The finding is simple: mHealth does not help. 

**Response: Thank you for your comment. Even when our study found that mHealth does not help, some study limitations could explain this finding. For that reason, we change our conclusion. We have edited our abstract conclusions (see response #2) and we have done the same in here.**

***“In our study, conducted in a low-income setting, the addition of mHealth was not effective in reducing foot ulceration or increasing adherence to thermometry after 18 months of follow-up. However, these results need to be interpreted with caution as the expected rates of DFU used in our sample size calculations were not met and there was a higher rate of previous DFU in the intervention group.”***

20. In the “comparison to previous studies”, the actual findings are not discussed. Part of this section is now a simple repetition of the introduction, the other parts fail to acknowledge the negative findings of the current study. [Why does mHealth appear to be beneficial in other studies, but not in this study? That is what should be reflected on. 

**Response: Thank you for your comment, some of the reasons that we cover in our discussion include:**

- **Most previous studies in mHealth were conducted in high-income countries with mostly young populations. In contrast, our study was implemented in a low-resource setting of a middle-income country with an elderly population. **
- **Outcomes in previous studies were glycated hemoglobin measurements or questionnaires and scales of self-management. We used ulceration, a patient-centered and clinically relevant outcome.**
- **The automated software system used in our study to send messages did not have bilateral communication, it was one-way only, and some studies suggest that bilateral communication may be a preferred route among people with diabetes.**
- **Finally, we are now including evidence from a recent systematic review of systematic reviews showing not so promising evidence from existing mHealth studies.**

 

“ ***A recent publication, evaluating 17 systematic reviews of mHealth intervention studies in diabetes and obesity ^8^, showed that fewer than half of the studies included in 2 reviews (out of 7 systematic reviews that covered the topic) improved diabetes management practices or medication adherence ^9, 10^ , and recommend the use of valid measures for outcomes and rigorous study designs to improve their quality”.***

21. I miss any discussion about the comment that alarms were not evaluated because of their low frequency. That implies that thermometry is not useful in ulcer prevention. This should be discussed in depth. 

**Response: As we clarified in response #10, the reporting of alarm signs to the study nurse was low, but information registered in the logbooks showed reports of alarm signs of up to 41%. We have now added this information in the results section (see response #10). Also, in the discussion we now have added the following statement:**

***“Also, in our results, 41% (44/108) of the study participants recorded alarm signs for two consecutive days in their logbooks, and we only have data from 9/44 (20%) that had a record of reporting an alarm sign to the study nurse. These figures do not consider those with alarm signs that did not seek nurse support or those who did report to the nurse but their report was not recorded.”***

***“The low rate of ulceration occurrence in our study could be potentially explained by two factors. First, that the participants did follow the instructions to reduce physical activity when observing alarm signs, even when they were not for two consecutive days or if they did not seek or receive the feedback of the study nurse. This is because the recommendations about reducing foot pressure and physical activity were given at the beginning of the study (videos) and they were also printed in their logbooks. Secondly, …”***

22. Ulcer incidence in patients with a previous ulcer is 27.8%, which is somewhat lower than other studies. Can this be attributed to the thermometer? And if so, how, if the thermometer never gives an alarm? Or is this because usual care is really good? 

**Response: We acknowledge that ulcer incidence was somewhat low in our study and perhaps the thermometry, provided to all study participants, may have been a contributing factor to the low incidence of ulcers observed. In our study, patients were explained about how to use the TempStat *™ *and one of the recommendations provided was to reduce physical activity if they find an alarm sign. Another practical feature was that the thermometer had a mirror in the middle of the two pads allowing the observation of the foot soles. So, it is possible that the thermometer alone was an effective intervention, in our population, for the prevention of the incidence of ulcers. Foot care among people with type 2 diabetes in Peru is very low, which also indicates need to enhance current standards of care in terms of prevention of foot ulcers.**

23. I miss discussion of the reliability and validity of the instrument, or the lack of knowledge thereof.

**Response: As mentioned before in the response #7, we have now added information about the reliability and validity of the TempStat *™***

24. In the limitation section, I miss acknowledging limitations in randomization (done with envelopes) and blinding (outcome assessor could be unblinded by patients). See comments in method section. 

**Response: We have already explained our randomization procedures (see response #12). Also, according to CONSORT guidelines, sealed envelopes are an acceptable method for randomization, and their explanation is as follow: “Enclosing assignments in sequentially numbered, opaque, sealed envelopes can be a good allocation concealment mechanism if it is developed and monitored diligently. This method can be corrupted, however, particularly if it is poorly executed. Investigators should ensure that the envelopes are opaque when held to the light, and opened sequentially and only after the participant’s name and other details are written on the appropriate envelope ^11,12^.**

Conclusion

25. Authors have a more clear conclusion in this section (first sentence). That is what should be used in the abstract and discussion. 

**Response: Yes, noted. We have edited our conclusion in the abstract (see response #2), and also edited our conclusion in the discussion based in the three reviewer’s suggestions, which now reads:**

***“In this randomized trial, conducted in a LMIC setting, the uptake of the foot thermometry for the prevention of foot ulcers was 87% in the intervention and control groups, and the addition of mHealth was not effective in reducing foot ulceration or increasing adherence to thermometry after 18 months of follow-up. However, these results need to be interpreted with caution as the expected rates of DFU used in our sample size calculations were not met and there was a higher rate of previous DFU in the intervention group."***

**References**

1.         Crawford F, Nicolson DJ, Amanna AE, et al. Preventing foot ulceration in diabetes: systematic review and meta-analyses of RCT data. *Diabetologia* 2020; **63**(1): 49-64.

2.         Skafjeld A, Iversen MM, Holme I, Ribu L, Hvaal K, Kilhovd BK. A pilot study testing the feasibility of skin temperature monitoring to reduce recurrent foot ulcers in patients with diabetes--a randomized controlled trial. *BMC Endocr Disord* 2015; **15**: 55.

3.         van Netten JJ, Sacco ICN, Lavery LA, et al. Treatment of modifiable risk factors for foot ulceration in persons with diabetes: a systematic review. *Diabetes Metab Res Rev* 2020; **36 Suppl 1**: e3271.

4.         van Netten JJ, Raspovic A, Lavery LA, et al. Prevention of foot ulcers in the at-risk patient with diabetes: a systematic review. *Diabetes Metab Res Rev* 2020; **36 Suppl 1**: e3270.

5.         Lazo-Porras M, Bernabe-Ortiz A, Sacksteder KA, et al. Implementation of foot thermometry plus mHealth to prevent diabetic foot ulcers: study protocol for a randomized controlled trial. *Trials* 2016; **17**(1): 206.

6.         Frykberg RG, Tallis A, Tierney E. Diabetic foot self examination with the TempStat™ as an integral component of a comprehensive prevention program. *The Journal of Diabetic Foot Complications* 2009; **1**(1): 13–8.

7.         Roback K, Johansson M, Starkhammar A. Feasibility of a thermographic method for early detection of foot disorders in diabetes. *Diabetes Technol Ther* 2009; **11**(10): 663-7.

8.         Wang Y, Min J, Khuri J, et al. Effectiveness of Mobile Health Interventions on Diabetes and Obesity Treatment and Management: Systematic Review of Systematic Reviews. *JMIR Mhealth Uhealth* 2020; **8**(4): e15400.

9.         de Ridder M, Kim J, Jing Y, Khadra M, Nanan R. A systematic review on incentive-driven mobile health technology: As used in diabetes management. *J Telemed Telecare* 2017; **23**(1): 26-35.

10.       Cotter AP, Durant N, Agne AA, Cherrington AL. Internet interventions to support lifestyle modification for diabetes management: a systematic review of the evidence. *J Diabetes Complications* 2014; **28**(2): 243-51.

11.       Schulz KF. Subverting randomization in controlled trials. *JAMA* 1995; **274**(18): 1456-8.

12.       CONSORT. 9. Randomisation: allocation concealment mechanism 2010. http://www.consort-statement.org/checklists/view/32--consort-2010/89-randomisation-allocation-concealment-mechanism (accessed 22th May 2020).
